# Genetic Polymorphisms in microRNA Genes Targeting PI3K/Akt Signal Pathway Modulate Cervical Cancer Susceptibility in a Chinese Population

**DOI:** 10.3389/fgene.2022.856505

**Published:** 2022-04-14

**Authors:** Kerong Chen, Zhiling Yan, Xudong Dong, Yan Liang, Yueting Yao, Shao Zhang, Weipeng Liu, Chuanyin Li, Yufeng Yao, Li Shi

**Affiliations:** ^1^ Institute of Medical Biology, Chinese Academy of Medical Sciences and Peking Union Medical College, Kunming, China; ^2^ Department of Gynaecologic Oncology, The No. 3 Affiliated Hospital of Kunming Medical University, Kunming, China; ^3^ The First People’s Hospital of Yunnan Province and The Affiliated Hospital of Kunming University of Science and Technology, Kunming, China; ^4^ College of Nursing Health Sciences, Yunnan Open University, Kunming, China

**Keywords:** MicroRNAs, Phosphatidylinositol 3 kinase, Signalling pathway, Single nucleotide polymorphisms, Association, Cervical cancer, Chinese population

## Abstract

Polymorphisms in microRNA (miRNA) genes could influence the expression of miRNAs that regulate the PI3K/Akt signalling pathway and play crucial roles in cancer susceptibility. To investigate the association of single nucleotide polymorphisms (SNPs) in miRNA genes of PI3K/Akt with cervical intraepithelial neoplasia (CIN) and cervical cancer (CC), nine SNPs located in miRNA genes were selected for genotyping, and the association of these SNPs with CIN and CC risk was evaluated. A total of 1,402 participants were enrolled in the current study, including 698 healthy individuals in the control group, 431 patients with CC, and 273 patients with CIN. Nine SNPs in miRNA genes (rs107822 in miR-219a, rs10877887 in let-7i, rs2292832 in miR-149, rs353293 in miR-143, rs3746444 in miR-499, rs3803808 in miR-132, rs4078756 in miR-10b, rs629367 in let-7a, and rs7372209 in miR-26a) were genotyped using MassArray, and the association of these SNPs with CIN and CC were analysed. The results showed that the frequencies of rs107822 in miR-219a and rs2292832 in miR-149 were significantly different between the control and CC groups (*p* < 0.005). The C allele of rs107822 in miR-219a was associated with an increased risk of CC (OR = 1.29, 95%CI:1.09–1.54) whereas the C allele of rs2292832 in miR-149 was associated with a decreased risk of CC (OR = 0.77, 95%CI:0.64–0.92). The results of inheritance model analysis showed that the best-fit inheritance models for rs107822 and rs2292832 were log-additive. The 2CC + CT genotype of rs107822 could be a risk factor for CC when compared with the TT genotype (OR = 1.28, 95%CI:1.08–1.51). The 2CC + CT genotype of rs2292832 could be a protective factor against CC when compared with the TT genotype (OR = 0.76, 95%CI:0.64–0.92). However, no association of these SNPs with CIN was found in the current study. Our results suggest that rs107822 in the promoter region of miR-219a and rs2292832 in pre-miR-149 region are associated with the risk of CC.

## Introduction

Cervical cancer (CC) is the fourth most common malignancy and the second most common gynaecological malignancy in women worldwide (Torre et al., 2015). It is predominantly caused by the persistent infection of high-risk human papilloma virus (HR-HPV) (Burd, 2003; zur Hausen, 2009). Malignant progression involves two main stages: cervical intraepithelial neoplasia (CIN) and CC, and occurs over a long period of time (more than 10 years) after HPV infection (Sasagawa et al., 2012).

The initiation and development of CC is also accompanied by aberrant regulation of host signalling pathways involving in essential cellular mechanisms (proliferation, invasion, survival, inflammation, and immunity), such as PI3K/Akt (Bossler et al., 2019). The PI3K/Akt signalling cascade regulates various fundamental aspects of cellular biology by promoting cell survival, growth, proliferation, migration, and energy metabolism (Morgensztern and McLeod, 2005; Sadeghi and Gerber, 2012; LoRusso, 2016; Aoki and Fujishita, 2017). The aberrant activation of PI3K/Akt signalling pathway has been found to be involved in various human cancers (Sharma et al., 2017; Ediriweera et al., 2019; Liu et al., 2020). In 2006, Bertelsen et al. reported PIK3CA amplification and increased Akt activation in cervical neoplasia (Bertelsen et al., 2006). In 2019, Zhang et al. found that PI3k/Akt/mTOR gene and protein levels increased in the CC tissues compared with the corresponding adjacent tissues (Zhang et al., 2019). Moreover, many studies have revealed by inhibiting or promoting PI3K signalling pathway, that genes could inhibit or promote the CC cells (Fu et al., 2020; Shi et al., 2020; Bai et al., 2021), these indicated the important roles of PI3K signalling pathway in CC.

Dysregulation of microRNAs (miRNAs) in human cancers highlights the important roles of these small single-stranded non-coding RNAs in human cancers (Garzon et al., 2006; Di Leva et al., 2014; Acunzo et al., 2015). They negatively regulate the expression of their target genes through the direct cleavage of mRNA or inhibition of mRNA translation, depending on the degree of complementarity between the seed sequence of miRNAs and their target UTR regions (Lai, 2002; Bartel, 2004; de Moor et al., 2005). Many studies have reported that miRNAs regulate components of the PI3K/Akt signalling pathway (Rahmani et al., 2020a; Rahmani et al., 2020b), and abnormal expression of these miRNAs might induce an out-of-control expression of their targets, which leads to disorders of the corresponding signalling pathway (Akbarzadeh et al., 2021). Studies have observed the abnormal expression of miR-219a (Xu et al., 2020), let-7i (Chhabra, 2018), miR-149 (Zhou and Xu, 2021), miR-143 (Tang et al., 2021), miR-132 (Zhang et al., 2021), miR-10b (Zou et al., 2016), let-7a (Wu et al., 2016) and miR-26a (Dong et al., 2014) in CC or other human cancers, which indicated the important roles of these miRNAs in human cancers. Single nucleotide polymorphisms (SNPs) in miRNA genes can modify the expression of mature miRNAs (Slezak-Prochazka et al., 2010; Króliczewski et al., 2018). Thus, SNPs in miRNA genes are associated with susceptibility to human cancers (Du et al., 2014; Wu et al., 2015; Wang et al., 2018). Previously, we found that rs4636297 in pri-miR-126 and rs11614913 in mature miR-196a2 were associated with CC risk (Yan et al., 2019), which indicates that SNPs in miRNAs might be associated with the development of CC.

In the current study, we first predicted potential targets of candidate miRNAs and enriched them in cancer signalling pathways. Next, miRNAs involved in the PI3K/Akt signalling pathway were screened. Finally, nine SNPs related to nine miRNA genes of PI3K/Akt (rs107822 in miR-219a, rs10877887 in let-7i, rs2292832 in miR-149, rs353293 in miR-143, rs3746444 in miR-499, rs3803808 in miR-132, rs4078756 in miR-10b, rs629367 in let-7a, and rs7372209 in miR-26a) were selected, and the association of SNPs with CIN and CC was evaluated in a Chinese population.

## Materials and Methods

### Subjects

A total of 273 patients with CIN and 431 with CC were recruited. The patients were diagnosed with CIN and CC at the Third Affiliated Hospital of Kunming Medical University from 2017 to 2019 according to “Diagnosis and Treatment: Obstetrics and Gynaecology” and the International Federation of Gynaecology and Obstetrics (FIGO 2009). The exclusion criteria for the study were as follows: 1) a prior history of primary cancer other than CC, 2) malignant tumours other than CC, 3) currently receiving radiotherapy or chemotherapy, and 4) an unclear diagnosis. According to the cervical pathological diagnostic criteria, CIN was classified into CIN I, II and III. CIN I is characterized as having slight atypical hyperplasia; CIN II as medium atypical hyperplasia; and CIN III as severe atypical hyperplasia (Schiffman et al., 2007). During the same period, 698 healthy women who underwent physical examinations at the same hospital were recruited as the control group. The genomic DNA of the samples was obtained from EDTA anti-coagulated whole blood of the subjects using QIAamp Blood Mini Kit (Qiagen NV, Venlo, Netherlands).

### Target Prediction and Signal Pathway Enrichment

The target genes of the miRNAs were predicted using the TargetScan Human 8.0 database (http://www.targetscan.org/vert_80/) (McGeary et al., 2019). Target enrichment was performed using the Database for Annotation, Visualization, and Integrated Discovery (DAVID) v6.8 (Huang et al., 2009).

### SNP Selection and Genotyping

First, the miRNAs involved in the regulation of the PI3K/Akt pathway were chosen through target prediction and enrichment. Then, SNPs which were located in the primary sequences, precursor sequences, or transcriptional regulatory regions of these miRNAs were selected. In addition, the MAF (minor allele frequency) of the SNPs was used as the selection criteria that only the SNPs with MAF over 0.05, were selected. As a result, nine SNPs (rs107822 in promotor region of miR-219a, rs10877887 in promotor region of let-7i, rs2292832 in pre-miRNA sequence of miR-149, rs353293 in promotor region of miR-143, rs3746444 in mature sequence of miR-499, rs3803808 in primary sequence region of miR-132, rs4078756 in promotor region of miR-10b, rs629367 in primary sequence region of let-7a, and rs7372209 in promotor region of miR-26a) were used. Information regarding the miRNA-SNPs selected in this study is presented in Table 1. Genotypes of the nine SNPs were determined using the Agena MassArray system. The PCR primers were designed using AssayDesigner 3.1 (Sequenom Inc., San Diego, CA, United States) (Supplementary Table S1). The PCR conditions and program have been described in our previous study (Li et al., 2020). A MALDI-TOF mass spectrometer (Agena, Inc, San Diego, CA, United States) was used to read SpectroCHIP, and the raw genotyping data was obtained using TYPER4.0 software. Samples were selected for sequencing to confirm the genotyping results for each SNP.

**TABLE 1:** The information of the nine SNPs selected in the current study.

SNPs	Genes	Function Consequence	Location	Alleles	MAF in EAS
rs107822	MIR219A	promotor region	Chr 6:33207798	T > C	0.396
rs10877887	MIRLET7I	promotor region	Chr 12:62603400	T > C	0.343
rs2292832	MIR149	pre-miRNA sequence	Chr 2:240456086	T > C	0.363
rs353293	MIR143	promotor region	Chr 5:149427663	C > T	0.156
rs3746444	MIR499	mature miR-499-5p	Chr20:34990448	A > G	0.145
rs3803808	MIR132	500bp Downstream	Chr17:2049683	A > G	0.455
rs4078756	MIR10B	promotor region	Chr2:176139387	T > C	0.271
rs629367	MIRLET7A	500bp Downstream	Chr11:122146306	A > C	0.219
rs7372209	MIR26A	promotor region	Chr3:37969217	C > T	0.272

### Statistical Analysis

Microsoft Excel software and the SPSS 19.0 statistical package were used for statistical analysis in the current study. The Hardy-Weinberg equilibrium (HWE) for each SNP in each group was evaluated. One-way analysis of variance (ANOVA) was used to compare the differences in age among the CIN, CC, and control groups. The differences in allele distributions of these SNPs in the CIN, CC, and control groups were analysed using Fisher’s chi-square test, and the odds ratios (ORs) with associated 95% confidence intervals (CIs) were calculated. Differences in the genotype distribution of these SNPs in the three groups were evaluated by inheritance model analysis using SNPstats software (Solé et al., 2006). The statistical power of the SNPs was calculated using “Power and sample size” software (Dupont and Plummer, 1990; Dupont and Plummer, 1998). The Bonferroni correction was performed for multiple comparisons, and the significance threshold was set at *p* < 0.005 (0.05/n, n = 9).

## Results

### Characteristics of the Subjects

A total of 1,402 participants were enrolled in this study. The general clinical characteristics of the participants are presented in Table 2. The average ages for the CIN, CC, and control groups were 46.80 ± 10.01, 47.74 ± 9.78, and 47.91 ± 7.18, respectively. No significant differences in age were found among the CIN, CC, and control groups (Table 2).

**TABLE 2:** The clinical characteristics of the subjects enrolled in the current study.

		CC	CIN	Control	*F*	*p* value
N		431	273	698		
Ages (year)		47.74 ± 9.78	46.80 ± 10.01	47.91 ± 7.18	1.662	0.190
Pathologic types	SCC (n)	359				
	AC(n)	53				
	Others (n)	19				
Stages of CC	I (n)	244				
	II (n)	157				
	III and IV (n)	30				
Stages of CIN	I (n)	71				
	II (n)	57				
	III (n)	145				

### Signal Pathway Enrichment of the miRNAs

**FIGURE 1:**
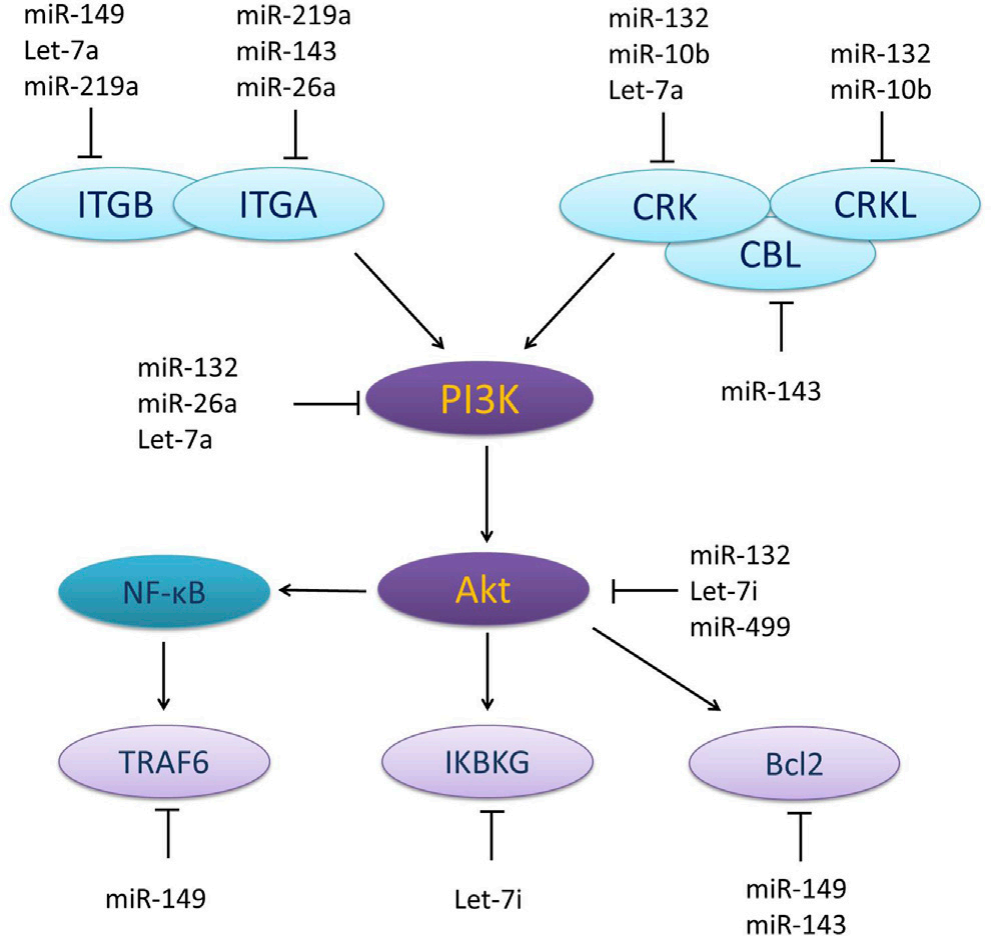
Nine miRNAs involving in PI3K/Akt signalling pathway.

Potential target genes of the miRNAs were predicted using TargetScan Human 8.0. The potential target genes were then submitted to DAVID to convert a gene list for enrichment. The enrichment results showed that nine miRNAs were involved in the PI3K/Akt signalling pathway (Figure 1).

### Association of the Alleles of the Nine SNPs With CIN and CC

The nine SNPs were all in HWE in the CIN, CC, and control groups (*p* > 0.05) (Table 3). The allelic distributions of the nine SNPs are presented in Table 4. The results showed that the allelic distribution of rs107822 in miR-219a and rs2292832 in miR-149 was significantly different between the CC and control groups (*p* = 0.004 and 0.004, respectively). The C allele of rs107822 in miR-219a was associated with an increased risk of CC (OR = 1.29, 95%CI:1.09–1.54). The C allele of rs2292832 in miR-149 was associated with a decreased risk of CC (OR = 0.77, 95%CI:0.64–0.92). No significant difference in the allelic distribution of the other SNPs was observed among the three groups (*p* > 0.005). And no association of all nine SNPs with CIN was found (*p* > 0.005).

**TABLE 3:** The Hardy–Weinberg equilibrium significance tests of the nine miRNA SNPs selected in the current study.

SNPs	Genotypes n (%)			HWE (*p-*value)
rs107822	T/T	T/C	C/C	
Control	275 (39.4)	314 (45.0)	109 (15.6)	0.221
CIN	101 (37.0)	124 (45.4)	48 (17.6)	0.355
CC	139 (32.3)	202 (46.9)	90 (20.9)	0.296
rs10877887	T/T	T/C	C/C	
Control	286 (41.0)	335 (48.0)	77 (11.0)	0.150
CIN	126 (46.2)	123 (45.1)	24 (8.8)	0.435
CC	185 (42.9)	200 (46.4)	46 (10.7)	0.457
rs2292832	T/T	T/C	C/C	
Control	293 (42.0)	316 (45.3)	89 (12.8)	0.792
CIN	114 (41.8)	132 (48.4)	27 (9.9)	0.207
CC	209 (48.5)	189 (43.9)	33 (7.7)	0.275
rs353293	C/C	C/T	T/T	
Control	499 (71.5)	180 (25.8)	19 (2.7)	0.570
CIN	181 (66.3)	84 (30.8)	8 (2.9)	0.640
CC	322 (74.7)	100 (23.2)	9 (2.1)	0.707
rs3746444	A/A	A/G	G/G	
Control	490 (70.2)	183 (26.2)	25 (3.6)	0.130
CIN	184 (67.4)	75 (27.5)	14 (5.1)	0.090
CC	292 (67.7)	119 (27.6)	20 (4.6)	0.088
rs3803808	A/A	A/G	G/G	
Control	246 (35.2)	340 (48.7)	112 (16.0)	0.761
CIN	83 (30.4)	134 (49.1)	56 (20.5)	0.887
CC	137 (31.8)	215 (49.9)	79 (18.3)	0.739
rs4078756	T/T	T/C	C/C	
Control	384 (55.0)	277 (39.7)	37 (5.3)	0.152
CIN	152 (55.7)	105 (38.5)	16 (5.9)	0.702
CC	250 (58.0)	155 (36.0)	26 (6.0)	0.762
rs629367	A/A	A/C	C/C	
Control	407 (58.3)	250 (35.8)	41 (5.9)	0.751
CIN	176 (64.5)	82 (30.0)	15 (5.5)	0.192
CC	255 (59.2)	145 (33.6)	31 (7.2)	0.105
rs7372209	C/C	C/T	T/T	
Control	323 (46.3)	294 (42.1)	81 (11.6)	0.262
CIN	117 (42.9)	126 (46.2)	30 (11.0)	0.651
CC	208 (48.3)	189 (43.9)	34 (7.9)	0.321

**TABLE 4:** The Allele distribution of the nine SNPs in control, CIN and CC groups.

SNPs	Alleles	Control n (%)	CIN n (%)	CC n (%)	CIN vs. control			CC vs. control	
					OR (95%CI)	*p* value		OR (95%CI]	*p* value
rs107822	T	864 (61.9)	326 (59.7)	480 (55.7)	1.10 (0.90–1.34)	0.374		1.29 (1.09–1.54)	0.004
	C	532 (38.1)	220 (40.3)	382 (44.3)					
rs10877887	T	907 (65.0)	375 (68.7)	570 (66.1)	0.85 (0.68–1.05)	0.121		0.95 (0.80–1.14)	0.575
	C	489 (35.0)	171 (31.3)	292 (33.9)					
rs2292832	T	902 (64.6)	360 (65.9)	607 (70.4)	0.94 (0.77–1.16)	0.583		0.77 (0.64–0.92)	0.004
	C	494 (35.4)	186 (34.1)	255 (29.6)					
rs353293	C	1,178 (84.4)	446 (81.7)	744 (86.3)	0.83 (0.64–1.07)	0.148		0.86 (0.67–1.09)	0.2241
	T	218 (15.6)	100 (18.3)	118 (13.7)					
rs3746444	A	1,163 (83.3)	443 (81.1)	703 (81.6)	0.86 (0.67–1.11)	0.255		0.89 (0.71–1.11)	0.285
	G	233 (16.7)	103 (18.9)	159 (18.4)					
rs3803808	A	832 (59.6)	300 (54.9)	489 (56.7)	0.83 (0.68–1.01)	0.062		0.89 (0.75–1.06)	0.179
	G	564 (40.4)	246 (45.1)	373 (43.3)					
rs4078756	T	1,045 (74.9)	409 (74.9)	655 (76.0)	1.00 (0.79–1.25)	0.981		0.94 (0.77–1.15)	0.546
	C	351 (25.1)	137 (25.1)	207 (24.0)					
rs629367	A	1,064 (76.2)	434 (79.5)	655 (76.0)	1.21 (0.95–1.54)	0.123		0.99 (0.81–1.21)	0.900
	C	332 (23.8)	112 (20.5)	207 (24.0)					
rs7372209	C	940 (67.3)	360 (65.9)	605 (70.2)	0.94 (0.76–1.16)	0.555		1.14 (0.95–1.38)	0.157
	T	456 (32.7)	186 (34.1)	257 (29.8)					

### Inheritance Model Analysis of the Nine SNPs With CIN and CC

Five inheritance models (codominant, dominant, recessive, overdominant, and log-additive) were analysed. Akaike Information Criterion (AIC) and Bayesian Information Criterion (BIC) values were used to determine the best-fit model, of which the AIC and BIC values were the lowest for each SNP (Solé et al., 2006). The association of the genotypes of the nine SNPs with CIN and CC was evaluated using inheritance model analysis (Tables 5, 6). The results showed that the genotypes of rs107822 and rs2292832 were significantly different between the CC and control groups (*p* = 4.6 × 10^−3^ and 0.004). The best-fit inheritance models for rs107822 and rs2292832 were log-additive. In this model, the 2CC + CT genotype of rs107822 was a risk factor for CC compared to the TT genotype (OR = 1.28, 95%CI:1.08–1.51). For rs2292832, the 2CC + CT genotype was a protective factor against CC compared with the TT genotype in this model (OR = 0.76, 95%CI:0.64–0.92). However, the results showed no association between the other SNPs and CIN or CC (*p* > 0.005).

**TABLE 5:** The inheritance model analysis of these SNPs between CIN and control groups.

SNPs	Model	Genotypes	Control (n%)	CIN (n%)	OR (95%CI)	*p* value	AIC	BIC
rs107822	Codominant	T/T	275 (39.4)	101 (37.0)	1.00	0.680	1,157.1	1,176.6
		C/T	314 (45.0)	124 (45.4)	1.07 (0.79–1.46)			
		C/C	109 (15.6)	48 (17.6)	1.20 (0.80–1.81)			
	Dominant	T/T	275 (39.4)	101 (37.0)	1.00	0.500	1,155.4	1,170.1
		C/T-C/C	423 (60.6)	172 (63.0)	1.10 (0.83–1.48)			
	Recessive	T/T-C/T	589 (84.4)	225 (82.4)	1.00	0.440	1,155.3	1,169.9
		C/C	109 (15.6)	48 (17.6)	1.16 (0.80–1.68)			
	Overdominant	T/T-C/C	384 (55.0)	149 (54.6)	1.00	0.930	1,155.9	1,170.5
		C/T	314 (45.0)	124 (45.4)	1.01 (0.76–1.34)			
	Log-additive	---	---	---	1.09 (0.90–1.33)	0.380	1,155.1	1,169.8
rs10877887	Codominant	T/T	286 (41.0)	126 (46.2)	1.00	0.320	1,155.6	1,175.1
		C/T	335 (48.0)	123 (45.0)	0.84 (0.62–1.12)			
		C/C	77 (11.0)	24 (8.8)	0.73 (0.44–1.21)			
	Dominant	T/T	286 (41.0)	126 (46.2)	1.00	0.160	1,153.9	1,168.5
		C/T-C/C	412 (59.0)	147 (53.9)	0.82 (0.62–1.08)			
	Recessive	T/T-C/T	621 (89.0)	249 (91.2)	1.00	0.360	1,155.1	1,169.7
		C/C	77 (11.0)	24 (8.8)	0.80 (0.49–1.30)			
	Overdominant	T/T-C/C	363 (52.0)	150 (55.0)	1.00	0.400	1,155.2	1,169.8
		C/T	335 (48.0)	123 (45.0)	0.89 (0.67–1.17)			
	Log-additive	---	---	---	0.85 (0.68–1.05)	0.130	1,153.6	1,168.3
rs2292832	Codominant	T/T	293 (42.0)	114 (41.8)	1.00	0.390	1,156.0	1,175.5
		T/C	316 (45.3)	132 (48.4)	1.07 (0.80–1.45)			
		C/C	89 (12.8)	27 (9.9)	0.78 (0.48–1.26)			
	Dominant	T/T	293 (42.0)	114 (41.8)	1.00	0.960	1,155.9	1,170.5
		T/C-C/C	405 (58.0)	159 (58.2)	1.01 (0.76–1.34)			
	Recessive	T/T-T/C	609 (87.2)	246 (90.1)	1.00	0.200	1,154.2	1,168.9
		C/C	89 (12.8)	27 (9.9)	0.75 (0.47–1.18)			
	Overdominant	T/T-C/C	382 (54.7)	141 (51.6)	1.00	0.380	1,155.1	1,169.8
		T/C	316 (45.3)	132 (48.4)	1.13 (0.86–1.50)			
	Log-additive	---	---	---	0.94 (0.76–1.16)	0.570	1,155.6	1,170.2
rs353293	Codominant	C/C	499 (71.5)	181 (66.3)	1.00	0.280	1,155.3	1,174.9
		C/T	180 (25.8)	84 (30.8)	1.29 (0.94–1.76)			
		T/T	19 (2.7)	8 (2.9)	1.18 (0.51–2.75)			
	Dominant	C/C	499 (71.5)	181 (66.3)	1.00	0.110	1,153.4	1,168.0
		C/T-T/T	199 (28.5)	92 (33.7)	1.28 (0.95–1.72)			
	Recessive	C/C-C/T	679 (97.3)	265 (97.1)	1.00	0.830	1,155.8	1,170.5
		T/T	19 (2.7)	8 (2.9)	1.10 (0.47–2.54)			
	Overdominant	C/C-T/T	518 (74.2)	189 (69.2)	1.00	0.120	1,153.5	1,168.1
		C/T	180 (25.8)	84 (30.8)	1.28 (0.94–1.74)			
	Log-additive	---	---	---	1.21 (0.94–1.58)	0.150	1,153.8	1,168.4
rs3746444	Codominant	A/A	490 (70.2)	184 (67.4)	1.00	0.460	1,156.4	1,175.9
		A/G	183 (26.2)	75 (27.5)	1.09 (0.79–1.50)			
		G/G	25 (3.6)	14 (5.1)	1.51 (0.77–2.98)			
	Dominant	A/A	490 (70.2)	184 (67.4)	1.00	0.390	1,155.1	1,169.8
		A/G-G/G	208 (29.8)	89 (32.6)	1.14 (0.85–1.54)			
	Recessive	A/A-A/G	673 (96.4)	259 (94.9)	1.00	0.270	1,154.7	1,169.3
		G/G	25 (3.6)	14 (5.1)	1.47 (0.75–2.88)			
	Overdominant	A/A-G/G	515 (73.8)	198 (72.5)	1.00	0.690	1,155.7	1,170.4
		A/G	183 (26.2)	75 (27.5)	1.07 (0.78–1.46)			
	Log-additive	---	---	---	1.15 (0.90–1.48)	0.260	1,154.6	1,169.3
rs3803808	Codominant	A/A	246 (35.2)	83 (30.4)	1.00	0.180	1,154.4	1,173.9
		G/A	340 (48.7)	134 (49.1)	1.18 (0.85–1.62)			
		G/G	112 (16.1)	56 (20.5)	1.47 (0.98–2.21)			
	Dominant	A/A	246 (35.2)	83 (30.4)	1.00	0.140	1,153.8	1,168.4
		G/A-G/G	452 (64.8)	190 (69.6)	1.25 (0.92–1.69)			
	Recessive	A/A-G/A	586 (83.9)	217 (79.5)	1.00	0.110	1,153.4	1,168
		G/G	112 (16.1)	56 (20.5)	1.34 (0.94–1.91)			
	Overdominant	A/A-G/G	358 (51.3)	139 (50.9)	1.00	0.870	1,155.9	1,170.5
		G/A	340 (48.7)	134 (49.1)	1.02 (0.77–1.36)			
	Log-additive	---	---	---	1.21 (0.99–1.48)	0.064	1,152.5	1,167.1
rs4078756	Codominant	T/T	384 (55.0)	152 (55.7)	1.00	0.930	1,157.8	1,177.3
		C/T	277 (39.7)	105 (38.5)	0.97 (0.72–1.29)			
		C/C	37 (5.3)	16 (5.9)	1.08 (0.58–1.99)			
	Dominant	T/T	384 (55.0)	152 (55.7)	1.00	0.880	1,155.9	1,170.5
		C/T-C/C	314 (45.0)	121 (44.3)	0.98 (0.74–1.30)			
	Recessive	T/T-C/T	661 (94.7)	257 (94.1)	1.00	0.780	1,155.8	1,170.4
		C/C	37 (5.3)	16 (5.9)	1.09 (0.60–2.00)			
	Overdominant	T/T-C/C	421 (60.3)	168 (61.5)	1.00	0.780	1,155.8	1,170.4
		C/T	277 (39.7)	105 (38.5)	0.96 (0.72–1.28)			
	Log-additive	---	---	---	1.00 (0.79–1.26)	0.990	1,155.9	1,170.5
rs629367	Codominant	A/A	407 (58.3)	176 (64.5)	1.00	0.210	1,154.8	1,174.3
		C/A	250 (35.8)	82 (30.0)	0.76 (0.56–1.03)			
		C/C	41 (5.9)	15 (5.5)	0.86 (0.46–1.60)			
	Dominant	A/A	407 (58.3)	176 (64.5)	1.00	0.084	1,152.9	1,167.5
		C/A-C/C	291 (41.7)	97 (35.5)	0.78 (0.58–1.04)			
	Recessive	A/A-C/A	657 (94.1)	258 (94.5)	1.00	0.870	1,155.9	1,170.5
		C/C	41 (5.9)	15 (5.5)	0.95 (0.52–1.75)			
	Overdominant	A/A-C/C	448 (64.2)	191 (70.0)	1.00	0.088	1,153.0	1,167.6
		C/A	250 (35.8)	82 (30.0)	0.77 (0.57–1.04)			
	Log-additive	---	---	---	0.84 (0.66–1.06)	0.140	1,153.7	1,168.3
rs7372209	Codominant	C/C	323 (46.3)	117 (42.9)	1.00	0.540	1,156.7	1,176.2
		T/C	294 (42.1)	126 (46.1)	1.18 (0.88–1.59)			
		T/T	81 (11.6)	30 (11.0)	1.03 (0.65–1.66)			
	Dominant	C/C	323 (46.3)	117 (42.9)	1.00	0.340	1,155.0	1,169.6
		T/C-T/T	375 (53.7)	156 (57.1)	1.15 (0.86–1.52)			
	Recessive	C/C-T/C	617 (88.4)	243 (89.0)	1.00	0.830	1,155.8	1,170.5
		T/T	81 (11.6)	30 (11.0)	0.95 (0.61–1.49)			
	Overdominant	C/C-T/T	404 (57.9)	147 (53.9)	1.00	0.270	1,154.7	1,169.3
		T/C	294 (42.1)	126 (46.1)	1.17 (0.88–1.55)			
	Log-additive	---	---	---	1.07 (0.87–1.31)	0.540	1,155.5	1,170.2

**TABLE 6:** The inheritance model analysis of these SNPs between CC and control groups.

SNPs	Models	Genotypes	Control n (%)	CC n (%)	OR (95%CI)	*p* value	AIC	BIC
rs107822	Codominant	T/T	275 (39.4)	139 (32.2)	1.00	0.018	1,501.2	1,521.4
		C/T	314 (45.0)	202 (46.9)	1.27 (0.97–1.67)			
		C/C	109 (15.6)	90 (20.9)	1.63 (1.15–2.31)			
	Dominant	T/T	275 (39.4)	139 (32.2)	1.00	0.015	1,501.4	1,516.5
		C/T-C/C	423 (60.6)	292 (67.8)	1.36 (1.06–1.76)			
	Recessive	T/T-C/T	589 (84.4)	341 (79.1)	1.00	0.026	1,502.3	1,517.4
		C/C	109 (15.6)	90 (20.9)	1.42 (1.05–1.94)			
	Overdominant	T/T-C/C	384 (55.0)	229 (53.1)	1.00	0.540	1,506.9	1,522.0
		C/T	314 (45.0)	202 (46.9)	1.08 (0.85–1.37)			
	Log-additive	---	---	---	1.28 (1.08–1.51)	4.6 x 10^−3^	1,499.0	1,514.0
rs10877887	Codominant	T/T	286 (41.0)	185 (42.9)	1.00	0.810	1,508.9	1,529.0
		T/C	335 (48.0)	200 (46.4)	0.92 (0.72–1.19)			
		C/C	77 (11.0)	46 (10.7)	0.93 (0.61–1.40)			
	Dominant	T/T	286 (41.0)	185 (42.9)	1.00	0.520	1,506.9	1,522.0
		T/C-C/C	412 (59.0)	246 (57.1)	0.92 (0.72–1.18)			
	Recessive	T/T-T/C	621 (89.0)	385 (89.3)	1.00	0.860	1,507.2	1,522.3
		C/C	77 (11.0)	46 (10.7)	0.97 (0.66–1.42)			
	Overdominant	T/T-C/C	363 (52.0)	231 (53.6)	1.00	0.600	1,507.0	1,522.1
		T/C	335 (48.0)	200 (46.4)	0.94 (0.74–1.19)			
	Log-additive	---	---	---	0.95 (0.79–1.14)	0.570	1,507.0	1,522.0
rs2292832	Codominant	T/T	293 (42.0)	209 (48.5)	1.00	0.009	1,499.9	1,520.0
		T/C	316 (45.3)	189 (43.9)	0.84 (0.65–1.08)			
		C/C	89 (12.8)	33 (7.7)	0.52 (0.34–0.80)			
	Dominant	T/T	293 (42.0)	209 (48.5)	1.00	0.033	1,502.7	1,517.8
		T/C-C/C	405 (58.0)	222 (51.5)	0.77 (0.60–0.98)			
	Recessive	T/T-T/C	609 (87.2)	398 (92.3)	1.00	0.006	1,499.8	1,514.9
		C/C	89 (12.8)	33 (7.7)	0.57 (0.37–0.86)			
	Overdominant	T/T-C/C	382 (54.7)	242 (56.1)	1.00	0.640	1,507.1	1,522.2
		T/C	316 (45.3)	189 (43.9)	0.94 (0.74–1.20)			
	Log-additive	---	---	---	0.76 (0.64–0.92)	0.004	1,499.0	1,514.0
rs353293	Codominant	C/C	499 (71.5)	322 (74.7)	1.00	0.460	1,507.7	1,527.8
		C/T	180 (25.8)	100 (23.2)	0.86 (0.65–1.14)			
		T/T	19 (2.7)	9 (2.1)	0.73 (0.33–1.64)			
	Dominant	C/C	499 (71.5)	322 (74.7)	1.00	0.230	1,505.9	1,520.9
		C/T-T/T	199 (28.5)	109 (25.3)	0.85 (0.65–1.11)			
	Recessive	C/C-C/T	679 (97.3)	422 (97.9)	1.00	0.500	1,506.8	1,521.9
		T/T	19 (2.7)	9 (2.1)	0.76 (0.34–1.70)			
	Overdominant	C/C-T/T	518 (74.2)	331 (76.8)	1.00	0.320	1,506.3	1,521.4
		C/T	180 (25.8)	100 (23.2)	0.87 (0.66–1.15)			
	Log-additive	---	---	---	0.86 (0.68–1.09)	0.210	1,505.7	1,520.8
rs3746444	Codominant	A/A	490 (70.2)	292 (67.8)	1.00	0.560	1,508.1	1,528.3
		A/G	183 (26.2)	119 (27.6)	1.09 (0.83–1.43)			
		G/G	25 (3.6)	20 (4.6)	1.34 (0.73–2.46)			
	Dominant	A/A	490 (70.2)	292 (67.8)	1.00	0.390	1,506.5	1,521.6
		A/G-G/G	208 (29.8)	139 (32.2)	1.12 (0.86–1.45)			
	Recessive	A/A-A/G	673 (96.4)	411 (95.4)	1.00	0.380	1,506.5	1,521.6
		G/G	25 (3.6)	20 (4.6)	1.31 (0.72–2.38)			
	Overdominant	A/A-G/G	515 (73.8)	312 (72.4)	1.00	0.610	1,507.0	1,522.1
		A/G	183 (26.2)	119 (27.6)	1.07 (0.82–1.41)			
	Log-additive	---	---	---	1.12 (0.90–1.39)	0.310	1,506.2	1,521.3
rs3803808	Codominant	A/A	246 (35.2)	137 (31.8)	1.00	0.400	1,507.4	1,527.5
		G/A	340 (48.7)	215 (49.9)	1.14 (0.87–1.49)			
		G/G	112 (16.1)	79 (18.3)	1.27 (0.89–1.81)			
	Dominant	A/A	246 (35.2)	137 (31.8)	1.00	0.230	1,505.8	1,520.9
		G/A-G/G	452 (64.8)	294 (68.2)	1.17 (0.91–1.51)			
	Recessive	A/A-G/A	586 (83.9)	352 (81.7)	1.00	0.320	1,506.3	1,521.4
		G/G	112 (16.1)	79 (18.3)	1.17 (0.86–1.61)			
	Overdominant	A/A-G/G	358 (51.3)	216 (50.1)	1.00	0.690	1,507.1	1,522.2
		G/A	340 (48.7)	215 (49.9)	1.05 (0.83–1.33)			
	Log-additive	---	---	---	1.13 (0.95–1.34)	0.170	1,505.4	1,520.5
rs4078756	Codominant	T/T	384 (55.0)	250 (58.0)	1.00	0.450	1,507.7	1,527.8
		C/T	277 (39.7)	155 (36.0)	0.86 (0.67–1.11)			
		C/C	37 (5.3)	26 (6.0)	1.08 (0.64–1.82)			
	Dominant	T/T	384 (55.0)	250 (58.0)	1.00	0.330	1,506.3	1,521.4
		C/T-C/C	314 (45.0)	181 (42.0)	0.89 (0.70–1.13)			
	Recessive	T/T-C/T	661 (94.7)	405 (94.0)	1.00	0.610	1,507.0	1,522.1
		C/C	37 (5.3)	26 (6.0)	1.14 (0.68–1.92)			
	Overdominant	T/T-C/C	421 (60.3)	276 (64.0)	1.00	0.210	1,505.7	1,520.8
		C/T	277 (39.7)	155 (36.0)	0.85 (0.67–1.10)			
	Log-additive	---	---	---	0.94 (0.77–1.15)	0.540	1,506.9	1,522.0
.rs629367	Codominant	A/A	407 (58.3)	255 (59.2)	1.00	0.580	1,508.2	1,528.3
		C/A	250 (35.8)	145 (33.6)	0.93 (0.72–1.20)			
		C/C	41 (5.9)	31 (7.2)	1.21 (0.74–1.97)			
	Dominant	A/A	407 (58.3)	255 (59.2)	1.00	0.780	1,507.2	1,522.3
		C/A-C/C	291 (41.7)	176 (40.8)	0.97 (0.76–1.23)			
	Recessive	A/A-C/A	657 (94.1)	400 (92.8)	1.00	0.380	1,506.5	1,521.6
		C/C	41 (5.9)	31 (7.2)	1.24 (0.77–2.01)			
	Overdominant	A/A-C/C	448 (64.2)	286 (66.4)	1.00	0.460	1,506.7	1,521.8
		C/A	250 (35.8)	145 (33.6)	0.91 (0.71–1.17)			
	Log-additive	---	---	---	1.01 (0.83–1.23)	0.900	1,507.3	1,522.3
rs7372209	Codominant	C/C	323 (46.3)	208 (48.3)	1.00	0.130	1,505.2	1,525.3
		T/C	294 (42.1)	189 (43.9)	1.00 (0.78–1.29)			
		T/T	81 (11.6)	34 (7.9)	0.65 (0.42–1.01)			
	Dominant	C/C	323 (46.3)	208 (48.3)	1.00	0.520	1,506.9	1,522.0
		T/C-T/T	375 (53.7)	223 (51.7)	0.92 (0.73–1.18)			
	Recessive	C/C-T/C	617 (88.4)	397 (92.1)	1.00	0.043	1,503.2	1,518.3
		T/T	81 (11.6)	34 (7.9)	0.65 (0.43–0.99)			
	Overdominant	C/C-T/T	404 (57.9)	242 (56.1)	1.00	0.570	1,506.9	1,522.0
		T/C	294 (42.1)	189 (43.9)	1.07 (0.84–1.37)			
	Log-additive	---	---	---	0.88 (0.73–1.05)	0.160	1,505.3	1,520.0

### Association Analysis of Nine SNPs With Different Pathological Types of CC

To investigate the association of the nine SNPs with the pathological types of CC, we analysed the distribution characteristics of the nine SNPs in different pathological types of CC. However, there were no significant differences in these SNPs between AC and SCC after Bonferroni correction (*p* > 0.005) (Supplementary Table S2).

### Association Analysis of Nine SNPs With Different Stages of CIN and CC

To investigate the association of the nine SNPs with different stages of CIN and CC, the CIN group was divided into CIN I + II and CIN III, and the CC group was divided into stages I and II + III + IV. No significant associations of these SNPs were observed between CIN I + II and CIN III and between CC stage I and stage II + III + IV after Bonferroni correction (*p* > 0.005) (Supplementary Tables S3, S4).

## Discussion

Alterations in the PI3K/Akt signalling pathway have been found in human cancers (Vara et al., 2004). These alterations might be a consequence of aberrant miRNA expression (Peng et al., 2019; Ichikawa et al., 2020). To explore the role of SNPs in miRNA genes involved in the PI3K/Akt pathway in CC susceptibility, the association of nine SNPs located in the miRNA genes involved in the PI3K/Akt pathway with CIN and CC was investigated. Results showed that the frequencies of rs107822 in miR-219a and rs2292832 in miR-149 were significantly different between the control and CC groups (*p* < 0.005).

To date, many studies have revealed that miR-219a functions as a tumour suppressor in different cancers, such as ovarian and breast cancer (Long et al., 2017; Xing et al., 2018; Wang et al., 2020; Ye et al., 2021). In the current study, we predicted that miR-219a could target integrins (ITGA and ITGB) which can participate in the activation of the PI3K/Akt signalling pathway. Moreover, our results showed that the rs107822C allele and CC genotype were risk factors for CC. Similarly, rs107822 has been reported to be associated with lung cancer (Zheng et al., 2017) and oesophageal squamous cell carcinoma (Song et al., 2015), and the C allele was associated with an increased risk of cancer. These results are consistent with those found in CC in the current study. Rs107822 is located at the 2 Kb upstream of miR-219a, which may be the transcriptional regulatory region of miR-219a. In 2012, Greliche et al. found that rs107822 in miR-219a could affect HLA-DPB1 expression in monocytes through interaction with rs1042448 in the 3′-UTR of *HLA-DPB1* (Greliche et al., 2012). The distance between rs107822 in miR-219a and rs1042448 in the 3′UTR of HLA-DPB1 is approximately 100 kb on chromosome 6, and these two SNPs show modest linkage disequilibrium (Greliche et al., 2012). Genome-wide association studies have revealed that loci susceptible for CC are located in the HLA-DP region (Chen et al., 2013; Shi et al., 2013), which indicates the important role of HLA-DP in CC. Thus, rs107822 may be associated with CC by affecting the expression of HLA-DPB1 through interaction with rs1042448 in the 3′UTR of *HLA-DPB1*. The interaction between rs107822 and the 3′UTR SNP (rs1042448) may be affected by miRNA expression (miRSNP) and miRNA binding specificity (3′UTR SNP) (Greliche et al., 2012). However, no association of this SNP with CIN was found in the current study, which was not consistent with the results of CC. As we known, the precancerous lesions and the carcinogenesis are different stages during the development of cervical cancer. Thus, one of the reasons of the discrepancy between CIN and CC could be miR-219a might play different roles in these two stages.

In 2020, Shao *et al.* reported that miR-149 functions as a tumour suppressor in CC by negatively regulating AURKA (Shao et al., 2020). Similarly, Zhou *et al.* found that miR-149 inhibits CC by targeting POU2F2 (Zhou and Xu, 2021). These results indicate a suppressive role of miR-149 in CC. In the current study, the results showed that rs2292832 was associated with CC susceptibility, and the C allele was associated with a decreased risk of CC. Our results are consistent with those of another study on CC by Wang et al. (2019). Similarly, the rs2292832 has been documented to be associated with various types of human cancer, such as gastric (Zhang et al., 2018), hepatocellular (Wang et al., 2014) and breast cancers (He et al., 2015). However, other studies have reported no such association (Dai et al., 2015; Li et al., 2016; Cîmpeanu et al., 2017; Yu et al., 2017). One of the reasons for the discrepancy between different studies is that rs2292832 may play different roles in different cancers. The other reason could be the different genetic background populations enrolled in the different studies. The third reason could be the different sample sizes in different studies which might affect the reliability of the association studies. Rs2292832 is located at the lower stream of the stem-loop structure of precursor miR-149, which might be related to the cleavage of pri-miRNA by DROSHA (Han et al., 2006; Auyeung et al., 2013). Thus, rs2292832 might be associated with CC through modulation of the maturation process of miR-149, subsequently affecting the expression of its target genes (ITGB and TRAF6) in the PI3K/Akt signalling pathway. Similar to rs107822, rs2292832 only exhibit an association with CC, not CIN, which might due to that miR-149 plays different roles between the precancerous lesions and the carcinogenesis stages in the cervical cancer development.

One limitation in the current study could be the lack of HPV status for our every patient, which makes it difficult to perform combined analyses of HPV status and gene SNPs interaction. Therefore, the roles of the interactions of HPV and host gene SNPs in the CC development should be investigated in the future.

## Conclusion

In summary, nine miRNAs involved in the PI3K/Akt signalling pathway were selected, and nine SNPs located in regions related to miRNA transcription or processing were chosen to investigate their association with CC. Our results showed that rs107822 of miR-219a and rs2292832 of miR-149 were associated with CC risk. The statistical power in the comparison between CC and control groups for rs107822 and rs2292832 were 0.818 and 0.803 respectively. Thus, the function of these two SNPs in the CC development should be investigated and verified in the future.

## Data Availability

The original contributions presented in the study are included in the article/Supplementary Material, further inquiries can be directed to the corresponding authors.
