# Supplementary material for: Genetic Polymorphisms in microRNA Genes Targeting PI3K/Akt Signal Pathway Modulate Cervical Cancer Susceptibility in a Chinese Population
Source: Front Genet. 2022 Apr 14;13:856505. doi: 10.3389/fgene.2022.856505 (PMC9047912; doi:10.3389/fgene.2022.856505)
Supplement: Supplementary file 1 [file DataSheet1.docx]

**Supplementary table 1 The primers used in MassArray for SNP genotyping**

| **SNPs** | **2nd-PCRP** | **1st-PCRP** | **UEP_SEQ** |
| --- | --- | --- | --- |
| rs107822 | ACGTTGGATGTATGTCCCTGTTTCCGCGTG | ACGTTGGATGTGCGTTTGGACAATCAGGAG | gggTCCGCGTGGTGTCTC |
| rs10877887 | ACGTTGGATGAAGAGAGAAAAGCGGCGGTG | ACGTTGGATGGCCCATTTCCTGCTTTCGAG | ggGGCGGTGTCCTGCGG |
| rs2292832 | ACGTTGGATGAACTCGCCCAGCCGGCCC | ACGTTGGATGTCTTCACTCCCGTGCTTGTC | cccctCGACCTGCGTTGTTCC |
| rs353293 | ACGTTGGATGTAATATCCCAAAAGGGCTCC | ACGTTGGATGAGGCTTTGGGAAGGGCTTCA | gggaCCAAAAGGGCTCCCCATTG |
| rs3746444 | ACGTTGGATGGGCTGTTAAGACTTGCAGTG | ACGTTGGATGACGGGAAGCAGCACAGACTT | aCCTCTCCACGTGAAC |
| rs3803808 | ACGTTGGATGTGAGGCCGTCCTGTCCTCC | ACGTTGGATGAATCGAAAGAACCCGAGTCC | ttcttTCCTCCCGGCCAAGGTATC |
| rs4078756 | ACGTTGGATGGGAGAAGCCATGAAGAACTC | ACGTTGGATGAAAGAGGGCATCTGTTCACG | aaacTTCAATTTAGGGTCACGTTT |
| rs629367 | ACGTTGGATGATTCTGTTTCCTCGGGTTAG | ACGTTGGATGTATGCAGCATTTTTGTGAC | TGAGGGATTCAGTATATCAG |
| rs7372209 | ACGTTGGATGCAGTCATGCTTACAGTCACG | ACGTTGGATGATGAGACAAAGGAGAGGCTG | gggagGCTTACAGTCACGTGGTAC |

**Supplementary table 2 The association of the SNPs with different pathological types of CC**

| SNPs | Alleles/  Genotypes | AC n(%) | SCC n(%) | OR[95%CI] | *P* value |
| --- | --- | --- | --- | --- | --- |
| rs107822 | T | 63(59.4) | 395(55.0) | 1.198[0.791-1.814] | 0.393 |
|  | C | 43(40.6) | 323(45.0) |  |  |
|  | T/T | 19(35.8) | 114(31.8) |  | 0.700 |
|  | T/C | 25(47.2) | 167(46.5) |  |  |
|  | C/C | 9(17.0) | 78(21.7) |  |  |
| rs10877887 | T | 72(67.9) | 475(66.2) | 1.083[0.700-1.676] | 0.719 |
|  | C | 34(32.1) | 243(33.8) |  |  |
|  | T/T | 26(49.1) | 151(42.1) |  | 0.342 |
|  | T/C | 20(37.7) | 173(48.2) |  |  |
|  | C/C | 7(13.2) | 35(9.7) |  |  |
| rs2292832 | T | 80(75.5) | 503(70.1) | 1.315[0.822-2.105] | 0.253 |
|  | C | 26(24.5) | 215(29.9) |  |  |
|  | T/T | 29(54.7) | 173(48.2) |  | 0.451 |
|  | T/C | 22(41.5) | 157(43.7) |  |  |
|  | C/C | 2(3.8) | 29(8.1) |  |  |
| rs353293 | C | 93(87.7) | 617(85.9) | 0.854[0.461-1.583] | 0.616 |
|  | T | 13(12.3) | 101(14.1) |  |  |
|  | C/C | 41(77.4) | 266(74.1) |  |  |
|  | C/T | 11(20.8) | 85(23.7) |  |  |
|  | T/T | 1(1.9) | 8(2.2) |  |  |
| rs3746444 | A | 89(84.0) | 583(81.2) | 0.825[0.475-1.432] | 0.493 |
|  | G | 17(16.0) | 135(18.8) |  |  |
|  | A/A | 40(75.5) | 239(66.6) |  | 0.123 |
|  | A/G | 9(17.0) | 105(29.2) |  |  |
|  | G/G | 4(7.5) | 15(4.2) |  |  |
| rs3803808 | A | 54(50.9) | 415(57.8) | 1.319[0.877-1.985] | 0.183 |
|  | G | 52(49.1) | 303(42.2) |  |  |
|  | A/A | 17(32.1) | 115(32.0) |  | 0.038 |
|  | A/G | 20(37.7) | 185(51.5) |  |  |
|  | G/G | 16(30.2) | 59(16.4) |  |  |
| rs4078756 | T | 81(76.4) | 544(75.8) | 1.036[0.641-1.674] | 0.884 |
|  | C | 25(23.6) | 174(24.2) |  |  |
|  | T/T | 31(58.5) | 208(57.9) |  | 0.978 |
|  | T/C | 19(35.8) | 128(35.7) |  |  |
|  | C/C | 3(5.7) | 23(6.4) |  |  |
| rs629367 | A | 74(69.8) | 548(76.3) | 1.394[0.890-2.184] | 0.146 |
|  | C | 32(30.2) | 170(23.7) |  |  |
|  | A/A | 25(47.2) | 215(59.9) |  | 0.187 |
|  | A/C | 24(45.3) | 118(32.9) |  |  |
|  | C/C | 4(7.5) | 26(7.2) |  |  |
| rs7372209 | C | 80(75.5) | 497(69.2) | 0.731[0.457-1.169] | 0.190 |
|  | T | 26(24.5) | 221(30.8) |  |  |
|  | C/C | 29(54.7) | 169(47.1) |  | 0.367 |
|  | C/T | 22(41.5) | 159(44.3) |  |  |
|  | T/T | 2(3.8) | 31(8.6) |  |  |

**Supplementary table 3 The association of the nine SNPs with the different clinical stages of CC**

| SNPs | Alleles/  Genotypes | I n(%) | II+III+IV n(%) | OR[95%CI] | P |
| --- | --- | --- | --- | --- | --- |
| rs107822 | T | 271(55.5) | 209(55.9) | 986[0.752-1.293] | 0.918 |
|  | C | 217(44.5) | 165(44.1) |  |  |
|  | T/T | 81(33.2) | 58(31.0) |  | 0.561 |
|  | T/C | 109(44.7) | 93(49.7) |  |  |
|  | C/C | 54(22.1) | 36(19.3) |  |  |
| rs10877887 | T | 330(67.6) | 240(64.2) | 1.166[0.878-1.549] | 0.289 |
|  | C | 158(32.4) | 134(35.8) |  |  |
|  | T/T | 109(44.7) | 76(40.6) |  | 0.535 |
|  | T/C | 112(45.9) | 88(47.1) |  |  |
|  | C/C | 23(9.4) | 23(12.3) |  |  |
| rs2292832 | T | 350(71.7) | 257(68.7) | 1.155[0.860-1.550] | 0.338 |
|  | C | 138(28.3) | 117(31.3) |  |  |
|  | T/T | 122(50.0) | 87(46.5) |  | 0.556 |
|  | T/C | 106(43.4) | 83(44.4) |  |  |
|  | C/C | 16(6.6) | 17(9.1) |  |  |
| rs353293 | C | 428(87.7) | 316(84.5) | 0.764[0.518-1.127] | 0.174 |
|  | T | 60(12.3) | 58(15.5) |  |  |
|  | C/C | 187(76.6) | 135(72.2) |  | 0.280 |
|  | C/T | 54(22.1) | 46(24.6) |  |  |
|  | T/T | 3(1.2) | 6(3.2) |  |  |
| rs3746444 | A | 401(82.2) | 302(80.7) | 0.910[0.644-1.286] | 0.593 |
|  | G | 87(17.8) | 72(19.3) |  |  |
|  | A/A | 168(68.9) | 124(66.3) |  | 0.855 |
|  | A/G | 65(26.6) | 54(28.9) |  |  |
|  | G/G | 11(4.5) | 9(4.8) |  |  |
| rs3803808 | A | 277(56.8) | 212(56.7) | 0.997[0.760-1.308] | 0.982 |
|  | G | 211(43.2) | 162(43.3) |  |  |
|  | A/A | 78(32.0) | 59(31.6) |  | 0.990 |
|  | A/G | 121(49.6) | 94(50.3) |  |  |
|  | G/G | 45(18.4) | 34(18.2) |  |  |
| rs4078756 | T | 378(77.5) | 277(74.1) | 1.203[0.879-1.647] | 0.248 |
|  | C | 110(22.5) | 97(25.9) |  |  |
|  | T/T | 149(61.1) | 101(54.0) |  | 0.287 |
|  | T/C | 80(32.8) | 75(40.1) |  |  |
|  | C/C | 15(6.1) | 11(5.9) |  |  |
| rs629367 | A | 372(76.2) | 283(75.7) | 0.670[0.708-1.329] | 0.848 |
|  | C | 116(23.8) | 91(24.3) |  |  |
|  | A/A | 143(58.6) | 112(59.9) |  | 0.519 |
|  | A/C | 86(35.2) | 59(31.6) |  |  |
|  | C/C | 15(6.1) | 16(8.6) |  |  |
| rs7372209 | C | 345(70.7) | 260(69.5) | 0.945[0.704-1.269] | 0.708 |
|  | T | 143(29.3) | 114(30.5) |  |  |
|  | C/C | 116(475) | 92(492) |  | 0.224 |
|  | C/T | 113(463) | 76(406) |  |  |
|  | T/T | 15(061) | 19(102) |  |  |

**Supplementary table 4 The association of the nine SNPs with different stages of CIN**

| SNPs | Alleles/  Genotypes | CIN I+II n(%) | CINIII n(%) | OR[95%CI] | P |
| --- | --- | --- | --- | --- | --- |
| rs107822 | T | 154(60.2) | 172(59.3) | 1.036[0.735-1.459] | 0.841 |
|  | C | 102(39.8) | 118(40.7) |  |  |
|  | T/T | 53(41.4) | 48(33.1) |  | 0.043 |
|  | T/C | 48(37.5) | 76(52.4) |  |  |
|  | C/C | 27(21.1) | 21(14.5) |  |  |
| rs10877887 | T | 184(71.9) | 191(65.9) | 1.325[0.920-1.908] | 0.131 |
|  | C | 72(28.1) | 99(34.1) |  |  |
|  | T/T | 65(50.8) | 61(42.1) |  | 0.300 |
|  | T/C | 54(42.2) | 69(47.6) |  |  |
|  | C/C | 9(7.0) | 15(10.3) |  |  |
| rs2292832 | T | 179(69.9) | 181(62.4) | 1.400[0.979-2.001] | 0.065 |
|  | C | 77(30.1) | 109(37.6) |  |  |
|  | T/T | 59(46.1) | 55(37.9) |  | 0.114 |
|  | T/C | 61(47.7) | 71(49.0) |  |  |
|  | C/C | 8(6.2) | 19(13.1) |  |  |
| rs353293 | C | 204(79.7) | 242(83.4) | 1.285[0.832-1.984] | 0.257 |
|  | T | 52(20.3) | 48(16.6) |  |  |
|  | C/C | 80(62.5) | 101(69.7) |  | 0.455 |
|  | C/T | 44(34.4) | 40(27.6) |  |  |
|  | T/T | 4(3.1) | 4(2.8) |  |  |
| rs3746444 | A | 204(79.7) | 239(82.4) | 1.195[0.778-1.835] | 0.416 |
|  | G | 52(20.3) | 51(17.6) |  |  |
|  | A/A | 85(66.4) | 99(68.3) |  | 0.405 |
|  | A/G | 34(26.6) | 41(28.3) |  |  |
|  | G/G | 9(7.0) | 5(3.4) |  |  |
| rs3803808 | A | 139(54.3) | 161(55.5) | 1.051[0.749-1.473] | 0.775 |
|  | G | 117(45.7) | 129(44.5) |  |  |
|  | A/A | 40(31.2) | 43(29.7) |  | 0.596 |
|  | A/G | 59(46.1) | 75(51.7) |  |  |
|  | G/G | 29(22.7) | 27(18.6) |  |  |
| rs4078756 | T | 202(78.9) | 207(71.4) | 1.500[1.012-2.224] | 0.043 |
|  | C | 54(21.1) | 83(28.6) |  |  |
|  | T/T | 81(63.3) | 71(49.0) |  | 0.054 |
|  | T/C | 40(31.2) | 65(44.8) |  |  |
|  | C/C | 7(5.5) | 9(6.2) |  |  |
| rs629367 | A | 192(75.0) | 242(83.4) | 1.681[1.105-2.556] | 0.015 |
|  | C | 64(25.0) | 48(16.6) |  |  |
|  | A/A | 71(55.5) | 105(72.4) |  | 0.008 |
|  | A/C | 50(39.1) | 32(22.1) |  |  |
|  | C/C | 7(5.5) | 8(5.5) |  |  |
| rs7372209 | C | 171(66.8) | 189(65.2) | 0.930[0.652-1.327] | 0.689 |
|  | T | 85(33.2) | 101(34.8) |  |  |
|  | C/C | 56(43.8) | 61(42.1) |  | 0.906 |
|  | C/T | 59(46.1) | 67(46.2) |  |  |
|  | T/T | 13(10.2) | 17(11.7) |  |  |
